# Plants used for making recreational tea in Europe: a review based on specific research sites

**DOI:** 10.1186/1746-4269-9-58

**Published:** 2013-08-13

**Authors:** Renata Sõukand, Cassandra L Quave, Andrea Pieroni, Manuel Pardo-de-Santayana, Javier Tardío, Raivo Kalle, Łukasz Łuczaj, Ingvar Svanberg, Valeria Kolosova, Laura Aceituno-Mata, Gorka Menendez-Baceta, Iwona Kołodziejska-Degórska, Ewa Pirożnikow, Rolandas Petkevičius, Avni Hajdari, Behxhet Mustafa

**Affiliations:** 1 Estonian Literary Museum, Vanemuise 42, Tartu 51003, Estonia; 2 Center for the Study of Human Health, Emory University, 550 Asbury Circle, Candler Library 107, Atlanta, GA 30322, USA; 3 University of Gastronomic Sciences, Piazza Vittorio Emanuele 9, I-12060, Pollenzo, Bra, Italy; 4 Departamento de Biología (Botánica), Universidad Autónoma de Madrid, c/Darwin 2, Campus de Cantoblanco, E-28049, Madrid, Spain; 5 Instituto Madrileño de Investigación y Desarrollo Rural, Agrario y Alimentario, Apdo. 127, 28800 Alcalá de Henares, Madrid, Spain; 6 Department of Food Science and Technology, Institute of Veterinary Medicine and Animal Sciences, Estonian University of Life Sciences, Kreutzwaldi 62, 51014 Tartu, Estonia; 7 Department of Botany and Biotechnology of Economic Plants; Institute of Applied Biotechnology and Basic Sciences, University of Rzeszów, Werynia 502, 36-100 Kolbuszowa, Poland; 8 Uppsala Centre for Russian and Eurasian Studies, Uppsala University, Box 514, SE-751 20 Uppsala, Sweden; 9 Institute for Linguistic Studies, Russian Academy of Sciences, Tuchkov pereulok 9, Saint-Petersburg 199053, Russia; 10 University of Warsaw Botanic Garden, ul. Ujazdowskie 4, 00-478 Warsaw, Poland; 11 Institute of Ethnology and Cultural Anthropology University of Warsaw ul., Żurawia 4, 00-503 Warsaw, Poland; 12 Department of Botany, Institute of Biology, University of Białystok, ul. Świerkowa 20 B, 15-950 Białystok, Poland; 13 Institute of Lithuanian Literature and Folklore, Antakalnio 6, Vilnius, Lithuania; 14 Department of Biology, University of Prishtina, St. Mother Teresa, Prishtinë, Kosovo

**Keywords:** Recreational tea, Social beverages, Local plants, Food culture, Tea consumption, *Origanum vulgare*

## Abstract

This paper is a review of local plants used in water infusions as aromatic and refreshing hot beverages (recreational tea) consumed in food-related settings in Europe, and not for specific medicinal purposes. The reviewed 29 areas are located across Europe, covering the post-Soviet countries, eastern and Mediterranean Europe. Altogether, 142 taxa belonging to 99 genera and 40 families were reported. The most important families for making herbal tea in all research areas were Lamiaceae and Asteraceae, while Rosaceae was popular only in eastern and central Europe. With regards to botanical genera, the dominant taxa included *Mentha*, *Tilia, Thymus, Origanum*, *Rubus* and *Matricaria*. The clear favorite was *Origanum vulgare* L., mentioned in 61% of the regions. Regionally, other important taxa included *Rubus idaeus* L. in eastern Europe, *Chamaemelum nobile* (L.) All. in southern Europe and *Rosa canina* L. in central Europe. Future research on the pharmacological, nutritional and chemical properties of the plants most frequently used in the tea-making process is essential to ensure their safety and appropriateness for daily consumption. Moreover, regional studies dedicated to the study of local plants used for making recreational tea are important to improve our understanding of their selection criteria, cultural importance and perceived properties in Europe and abroad.

## Definition of the study object

We propose to use the term “recreational tea” in the paper to describe those herbal beverages prepared as infusions and that are consumed in a food context for their general social and/or recreational value or for their general attributions of being “healthy” drinks. This definition excludes those teas prepared and consumed only for specific medicinal purposes.

## Introduction

Although the English term “tea” denotes the infusion made of the leaves of *Camellia sinensis* (L.) Kuntze, it also refers in colloquial language to the wide variety of locally grown herbs used in different regions of the world for recreational tea.

In this article, we use recreational tea as a technical term for an infusion made of leaves or flowers of taxa other than *C. sinensis*. Such beverages were already known in Europe long before the oriental tea was introduced there in 1606 by the Dutch East India Company [1]. However, they have also been used as substitutes for the oriental tea. Many of these plants have folk names like 'tea-leaves’ and 'tea-plants’ in various native languages [2].

Historically, some people have shown a preference for recreational tea although they could afford the “real thing”. Recall Agatha Christie’s fictional character Hercule Poirot who always drank recreational tea. The medicinal properties of the infusions of local plants were well known and prized by most herbalists, but it is difficult to state that the habit of drinking herbal tea as an accompaniment to one’s meal or as a social activity was a common practice before the introduction of the oriental tea. Nevertheless, as there is a growing interest in research on the chemical composition of specific herbal teas produced commercially in different regions of the world (e.g., see [3-5]) there is also the need for comparative ethnobotanical research on the plants used for making food-side infusions in different areas of the world. Although a few regional studies on European teas have already been published [6-8], most reports list only a few plants for making tea among the food plants of a specific region (e.g., see [9-13]).

Our research contributes to the European chapter of the worldwide review on the use of local plants for making tea. Our main objective was to assess and compare the available information on plants used for recreational tea purposes in continental Europe. We argue that despite the fact that a wide variety of plants are used in different regions, only a few specific genera or even species are preferred as the source for making infusions used in the context of food, and not for specific medicinal properties.

## Data and methods

**Figure 1:**
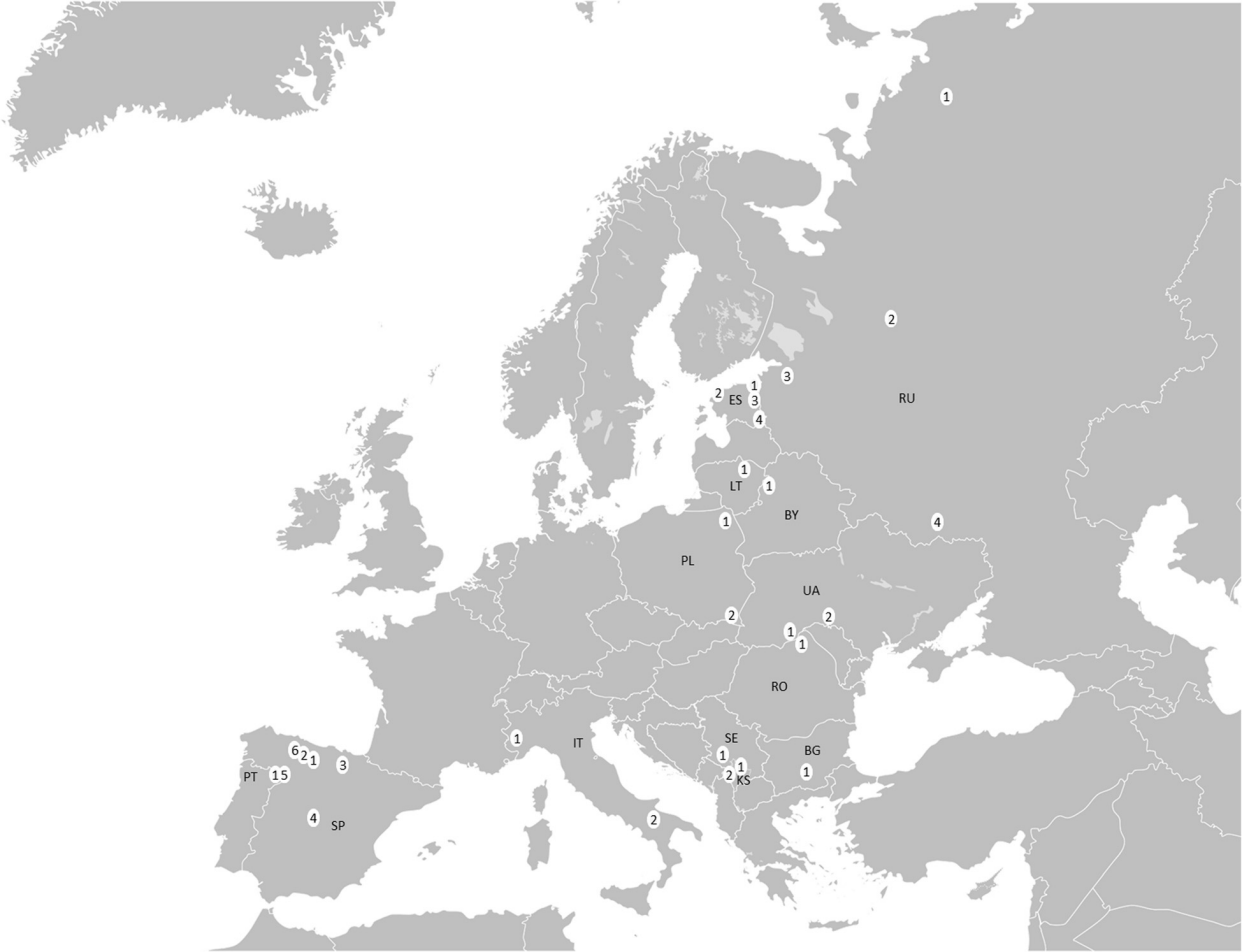
**The map of the regions covered by the review.** Map base: http://upload.wikimedia.org/wikipedia/commons/5/5a/BlankMap-Europe-v4.png.

This review relies on numerous ethnobotanical studies and published ethnographies as well as unpublished fieldwork results. Although there are many historical sources that reflect on the use of local species for food, the authors were not aiming to cover them all, as the identification of the species listed in historical sources can oftentimes be problematic (see [14]). Instead, we selected 29 sample regions located in 14 countries, covering mostly post-Socialist countries (Russian Federation, Estonia, Lithuania, Ukraine, Belarus, Poland, Bulgaria, Romania, Kosovo, Serbia) and Mediterranean countries (Italy, Spain and Portugal). The geographical distribution of the regions is denoted in Figure 1. The period of data collection for the studies included in our review ranges from 1926 to 2012. Detailed information on each study region is presented in Table 1.

**Table 1:** Characteristics of the regions and field studies included in our review

**Reg.**	**State**	**Region**	**Year**	**N**	**RN**	**UR**	**CI**	**SP**	**Age**	**Landscape**	**Language**	**Occup.**	**Method**	**Clim.**	**Source**
**RU1**	Russian Federation	Ust’-Tsil’ma region of Komi Republic	2001	nk	nk	nk		2	nk	Paldual meadows, fir forests	Russian, Komi	F	nk	Dfc	[15]
RU2*	Russian Federation	Vologda	1990s	nk	nk	nk		5	nk	Shallow, decidous and conifer forests	Russian	A	nk	Dfc	[16]
RU3*	Russian Federation	St Petersburg	2000s	nk	nk	nk		6	nk	Taiga, mixed forests,	Russian	M	SB	Dfc	Exp. RU1
**RU4**	Russian Federation	Belgorod oblast	1926	nk	nk	nk		4	nk	Hilly meadows, decidous forests	Russian	A	nk	Dfb	[17]
**ES1**	Estonia	Kohla-Järve	1930	27	10	34	1.26	17	45-80	Costal line, meadows, conifer forests	Estonian	A	HA	Dfb	[18]
**ES2**	Estonia	Kullamaa	1930	32	17	49	1.53	14	45-80	Costal line, wooden meadows, meadows, decidous and conifer forests	Estonian	A	HA	Dfb	[18]
**ES3**	Estonia	Peipsi	1999-2008	46	11	31	0.67	11	61	Shore of the large lake, conifer forests	Russian	M	I, PO, SB	Dfb	[19]
**ES4**	Estonia	Räpina	1930	29	11	56	1.93	16	45-80	Flat inland, meadows, conifer forests,	Seto	A	HA	Dfb	[18]
**UA1**	Ukraine	Storozhinets region of Chernovtsy oblast’	1999-2000	nk	59	14		8	nk	Broadleaf forest, mountainous pastures, flat cornfields	Ukrainian, Romanian	A	SB	Dfb	Exp UA1
**UA2**	Ukraine	Strointsy, Tivriv region, Vinnitsa oblast’, Ukraine	2012	47	47	nk		29	55	Broad leaf forest, steppe	Ukrainian	A	I, SS, PO, SB	Dfb	Exp UA2
**LT1**	Lithuania	Užpaliai district	2010	33	33	23	0.70	16	44-90	meadows, forest	Lithuanian	M	I, SS	Dfb	[20]
**BY1**	Belarus	Gervėčiai ethnic region	2010	62	62	61	0.98	17	40-91	meadows, forest	Lithuanian, Russian, Belarusian	A	I, SS	Dfb	[21]
**PL1**	Poland	Puszcza Knyszyńska	2006-2012	89	68	248	2.79	37	65	hilly, mixed forests	Polish	A	SB	Dfb	[22]
**PL2**	Poland	Pogórzanie ethnogrphic region (Krosno and Jasło area), SE Poland	2010 + PO 1975-2012	133 PO	nk	nk		8	69	hilly, mixed forests	Polish	M	SB, PO	Dfb	[23,24]
**RO1**	Romania	Bukovina Pojana Mikuli (Poiana Mikului)	2005-2006	28	28	94	3.36	10	48	mountainous, beech forest	Polish	M	I, SS, PO, SB	Dfb	[25] + Exp RO1
**BG1**	Bulgaria	Chepelare community, Smoljan region	2007	nk	9	37		22	nk	mountainous mixed forest, small agricultural flatlands and meadows	Bulgarian	M	SB	Dfc	Exp BG1
**BG2**	Bulgaria	Laki community, Asenovgrad region	1992-1999	nk	28	9		9	nk	mountainous mixed forest, small agricultural flatlands and meadows	Bulgarian	M	SB	ET	Exp BG2
**KS1**	Kosovo	Gollak	2009	66	29	nk		9	>50	hilly, mixed forests	Albanian	M	SB	Dfb	[26]
**KS2**	Kosovo	Albanian Alps	2010	91	30	nk		12	50-79	hilly, mixed forests	Albanian	M	SB	Dfb	[27]
**SE1**	Serbia	Pester Plateau	2010	nk	42	nk		22	43-93 years old	pasture and meadows	Serbo-Croatian	A	SB	Cfa	[28]
**IT1**	Italy	Western Italian Alps	2011	81	nk	nk		8	mid-aged & elderly	mountains	Alpine Provencal & Kye	A	SB	Cfa	[29]
**IT2**	Italy	Vulture Alto Bradano	2000-2001	44	nk	nk		21	47-94 years old	hilly, mixed meadow & forest	Italian	M	SB	Csb	[30]
**SP1**	Spain	Campoo	1999-2001	107	nk	45	0.42	9	68	mosaic of meadows and forests, and high mountain vegetation	Spanish	M	SB	Cfb	[31-33]
**SP2**	Spain	Piloña	1999-2003	94	nk	36	0.38	9	57	mosaic of meadows and forests, and high mountain vegetation	Spanish	M	SB	Cfb	[34-36]
**SP3**	Spain	Gorbeialdea	2008-2010	103	2	2	0.02	1	74	mountainous: pastures mixed with Pinus radiata plantations and forests	Basque	I, S	SB	Cfb	[37]
**SP4**	Spain	Sierra Norte de Madrid	2003-2009	112	52	82	0.73	17	68	mountainous mixed forest agricultural valleys and pastures	Spanish	S	SB	Bsk	[38]
**SP5**	Spain	Sanabria	2004?	44	nk	11	0.25	5	nk	mosaic of meadows and forests, and high mountain vegetation	Spanish	nk	SB	Csb	[39]
**SP6**	Spain	Picos de Europa	nk	131	nk	96	0.73	6	nk	mountainous: mosaic of meadows and forests, and high mountain vegetation	Spanish	M	SB	Cfb	[31,40]
**PT1**	Portugal	Montesinho	2000-2004	107	nk	293	2.74	21	62	mosaic of meadows and forests and high mountain vegetation	Portuguese	M	SB	Csb	[31,34,41,42]

Abbreviations: *Reg* abbreviation for region, *Year* year of research or publication, *N* number of study participants, *RN* number of respondents reporting the use of plants as recreational tea, *UR* nr of use-reports for recreational teas, *CI* cultural importance of the category, i.e., UR/N, *SP* nr of species used as recreational tea, *Age* range or average age of respondents, *Landscape* landscape type, *Language* dominant language in the region, *Occup* primary occupation in the region (*A* agrarian, *F* forestry, *I* industry, *M* mixed, *S* service), *Clim* climate of the region according to the Köppen-Geiger climate classification system [43] (*Bsk* cold semi-arid climate, *Cfa* warm oceanic climate/humid subtropical climate, *Cfb* temperate oceanic climate, *Csb* temperate Mediterranean, *Dfb* temperate continental climate/humid continental climate, *Dfc* cool continental climate/subarctic climate, *ET* tundra climate), *Method* fieldwork method (*HA* homework assignment for schoolchildren, *I* interview, *PO* participant observation, *SB* snowball sampling, *SS* semi-structured questionnaire); *nk* not known, * - local plants are used as additives to oriental tea.

In this review, we included only those species that are collected by people from local wild populations or those which are cultivated in home gardens for personal or family use. The qualitative data set from Scandinavia was included in this review only as a point of comparison.

As the number of recent field studies on this topic in eastern Europe is limited, we also included some archival sources in our analysis. The identification of plant taxa originating from archival sources and ethnographic publications was made according to the following algorithm: 1) Latin name, if provided; 2) regional name; 3) generally common name; or 4) a combination of any of these. Botanical nomenclature follows nomenclature set forth in *The Plant List*[44]. If the plant was not identifiable at least to the level of genus, it was not considered in our analysis.

Information concerning the use of local plants for making recreational herbal teas has typically been collected as ancillary data in ethnobotanical or ethnographic field studies that are otherwise focused on the documentation of traditional knowledge and use of medicinal species, edible plants, or other general uses of plants. Although all contemporary conducted field studies discussed in the article had at least one of the article’s authors as a participant and special effort was made to obtain a high level of detail concerning the regional characteristics and the research methods used for the various studies, in some cases, some information was missing. This was denoted as “nk”, or not known, in Table 1.

As different methodologies for data collection were employed in the studies reviewed here, it was not possible to complete a comparative statistical analysis. Thus, we instead performed a semi-quantitative analysis and used an Excel database to analyze the complete dataset, comprising information gleaned from all of the reviewed studies. The most popular taxa were selected based on the level of species, genera and family.

To improve our understanding of regional importance of the most popular taxa, the use-reports [45], when available, were also included along with the number of regions where the plants were used in the context of recreational teas. When possible, the cultural importance index of this use-category was calculated [37,38,45]. This is a useful indicator for comparing the cultural value of recreational teas in the different regions. Greater values of this index will be found in the regions where these herbal teas have a greater importance. For detailed list on the taxa used in every region see [Additional file 1].

## Results and discussion

Here, we have divided our assessment of the data into sections based on taxonomic level (species, genera, and families), regional differences and similarities, and considerations concerning perceived health value of the most important flora used in the recreational tea context.

As can be seen in Additional file 1, in the 29 different regions of Europe studied, 142 taxa were used for making recreational herbal teas. Table 2 shows the list of the 21 species whose use was mentioned in more than three regions. In addition, among the taxa listed in at least four regions, six were generalizations that were only identified to the genus level. Another 16 taxa were listed in three regions, 27 in two and 72 taxa only in one.

**Table 2:** Most frequently mentioned species and their regional distributions

**Family**	**Scientific plant name**	**Regions**	**Total use-reports**	**Areas**	**Parts used**
Lamiaceae	*Origanum vulgare* L.	18	41	All	aerial parts
Rosaceae	*Rubus idaeus* L.	11	56	EE	twigs, aerial parts, fruits, leaves
Lamiaceae	*Thymus serpyllum* L.	10	33	EE	aerial parts
Hypericaceae	*Hypericum perforatum* L.	9	38	EE, CE	aerial parts
Asteraceae	*Achillea millefolium* L.	8	27	All	inflorescences, aerial parts, roots
Rosaceae	*Rosa canina* L.	8	22	EE, CE	flowers, leaves, peels, fruits
Asteraceae	*Chamaemelum nobile* (L.) All.	7	104	SE	inflorescences
Apiaceae	*Carum carvi* L.	7	49	EE, CE	seeds, aerial parts
Rosaceae	*Fragaria vesca* L.	7	30	EE	flowers, leaves, fruits, aerial parts
Lamiaceae	*Thymus pulegioides* L.	7	21	CE, SE	aerial parts
Rosaceae	*Malus domestica* Borkh*.*	7	17	EE, CE	flowers, leaves, peels, fruits, one year old twigs
Ericaceae	*Vaccinium myrtillus* L*.*	7	16	EE, CE	aerial parts, fruits, flowers, leaves
Lamiaceae	*Melissa officinalis* L.	6	56	All	flowering aerial parts
Adoxaceae	*Sambucus nigra L.*	6	24	CE	flowers
Lamiaceae	*Mentha x piperita* L.	5	28	All	aerial parts
Lamiaceae	*Mentha longifolia* (L.) L.	5	15	CE	aerial parts
Tiliaceae	*Tilia cordata* Mill.	5	5	All	bark and complete inflorescence, including the bract that makes the fruits fly
Lamiaceae	*Mentha pulegium* L.	4	29	CE	flowering shoots
Ericaceae	*Vaccinium vitis-idaea* L.	4	7	EE, CE	flowers, aerial parts, fruits
Asteraceae	*Matricaria chamomilla* L.	4	5	CE, SE	flowering aerial parts
Rosaceae	*Prunus cerasus* L.	4	4	EE	leaves, flowers, one year old twigs

Abbreviations: *EE* eastern Europe, *CE* central Europe, *SE* southern Europe.

### Species

The majority of the top 10 species are well known in European folk medicine for their digestive properties, which is also one of the reasons cited for the selection of plants for teas to accompany meals [6,7]. In addition, many of the same top species are also perceived as having anti-inflammatory properties, such as *Thymus serpyllum* L., *Achillea millefolium* L. and Chamaemelum nobile (L.) All. [46].

All of these species are also named in many scientific and popular publications as possible or regional tea substitutes. The taxon used in more than half of the selected regions, *Origanum vulgare* L.*,* was also mentioned centuries ago by Carl Linnaeus [47]. Likewise, *Carum carvi* L. was used in Sweden for making tea already in the mid-18th century [48] and *Thymus praecox* Opiz was known as a tea substitute on the Faroes in the 19th century [49,50]. The recreational tea use of *Sambucus nigra* L. was mentioned in 1765 in Sweden [13].

### Genera

As the territory covered by the regions under study is considerably large, not all of the species grow everywhere, even under cultivation. Therefore, the most reliable way to detect the most important plants in the tea consumption of Europe is to find the most used genera in all of the study regions. Altogether, 99 genera are represented and 18 of them were cited in at least five regions (Table 3).

**Table 3:** Most frequently mentioned genera represented by at least two species

**Family**	**Genera**	**Regions**	**Identified species**	**Total UR**	**Areas**
Lamiaceae	*Mentha*	22	6	191	All
Lamiaceae	*Origanum*	19	2	42	All
Tiliaceae	*Tilia*	18	2	142	All
Lamiaceae	*Thymus*	17	4	59	All
Rosaceae	*Rubus*	11	3	60	EE, CE
Asteraceae	*Matricaria*	13	2	59	All
Hypericaceae	*Hypericum*	10	2	39	EE, CE
Rosaceae	*Malus*	9	2	25	EE, CE
Ericaceae	*Vaccinium*	8	3	25	EE, CE
Rosaceae	*Rosa*	8	2	26	EE, CE
Adoxaceae	*Sambuccus*	8	1	26	EE, CE
Lamiaceae	*Melissa*	8	1	12	EE, CE
Rosaceae	*Crataegus*	7	2	9	CE
Rosaceae	*Prunus*	6	2	9	EE, CE
Grossulariacea	*Ribes*	5	2	12	EE, CE
Gentianaceae	*Centaurium*	5	1	9	CE
Primulaceae	*Primula*	5	1	14	EE
Fabaceae	*Trifolium*	5	2	6	EE

Abbreviations: *EE* eastern Europe, *CE* central Europe, *SE* southern Europe.

The domination of *Mentha* as a highly valued genus is not surprising: its wide international use has been reported [12] and different commercial versions of it have been sold worldwide for several centuries already. Quite expectedly, the list of the most popular genera contains the majority of the most popular species. Still, the list contains a few more genera with high importance: *Ribes, Crataegus, Trifolium, Primula* and *Centaurium*. The majority of these were absent from the species list most probably due to difficulties in differentiating them on the species level in many sources.

### Families

Preference for certain plant families is also of equal importance, as this allows for the future comparison of the most used European plants with those used internationally. Altogether, 40 families were represented in the list of the cited species. Representatives of 12 of them were mentioned in more than 5 regions (Table 4).

**Table 4:** Frequency of family citations containing more than one species

**Family**	**Regions**	**Identified species**	**Total UR**	**Areas**
Lamiaceae	27	26	446	All
Asteraceae	24	22	278	All
Rosaceae	18	19	168	EE, CE
Tiliaceae	18	2	143	All
Apiaceae	10	4	79	All
Hypericaceae	10	2	39	EE, CE
Ericaceae	8	4	26	EE, CE
Adoxaceae	8	2	27	CE
Fabaceae	8	4	18	All
Grossulariaceae	5	2	12	EE, CE
Gentianaceae	5	1	10	EE, CE
Boraginaceae	5	3	7	All

Abbreviations: *EE* eastern Europe, *CE* central Europe, *SE* southern Europe.

Among the families included in our list, only three are represented with a considerable number of species: The most popular family is Lamiaceae (26 identified species), followed by Asteraceae (22 identified species) and Rosaceae (19 identified species). While the use of Lamiaceae and Asteraceae is spread across Europe, the cited members of the Rosaceae are well-known as local herbal teas only in eastern and central Europe. The parallel could be drawn here to the popularity of the species of Rosaceae and Asteraceae families as wild food plants in eastern Europe [51,52], but also as medicinal plants in different parts of Europe; while plants from the Lamiaceae family have been most important in seasoning and making beverages [53].

Although the importance of Tiliaceae in European food culture has been demonstrated already in Tables 2 and 3, its leading position among the families with just a few species represented is of crucial importance. In Marcel Proust’s famous novel *À la recherche du temps perdu* (1913), the author was overwhelmed by memories while dipping madeleines in linden-tea. In the beginning of 20th century *Tilia* was not used for making even medicinal infusions in Estonia [7,54]. Still, in Polish settlements in Romania, the inflorescences of *Tilia* are used as the “main” tea component, to which other species were added [25].

### Regional differences

The regions selected for this review have been divided into three geographic areas in Europe: East, Central and South. Observing the results of the case-studies included in the tables, we can appreciate two main differences. Firstly, we can compare differences in the cultural importance of recreational teas among the studied regions. Though there are many regions without available data, there seem to be great differences in the cultural importance (CI) of this use-category among these regions. Greater values, and thus greater cultural importance of recreational teas, are found in some studies from eastern Europe (Romania, Poland, Estonia), while the lowest values are found in some Iberian regions (i.e. Basque Country and Sanabria).

Secondly, there are many regional differences concerning the plants used for making recreational tea. In fact, only a few species are used throughout all of Europe, with the majority being used only regionally. This could be attributed to differences in climate and habitat that influence the growth of plants, but also to cultural attitudes towards recreational tea in the respective study regions. For example, in places like Iceland and the Faroe Islands, very few taxa were actually available and therefore inhabitants had to use native plants that are commonly found in the landscape. In Iceland, *Dryas octopetala* L. has been used [50]. The Saami in Norway and Sweden made an infusion of the bracket fungus *Piptoporus betulinus* (Bull. ex Fr.) P. Karst., which seems to have been common before coffee was introduced in the 1860 s. During World War II, when there was a shortage of imported foods, there was a revival of using birch bracket for making a hot drink among the Saami. This bracket fungus has until recently been made into a drink by Saami children in Norway [13].

Related to the use reports (UR) and the CI, there is also a considerable difference in the actual number of plant taxa reported in each region (see Table 1). While the mean number of the reported taxa is 12.8, the range of the reports goes from 1 to 37 taxa reported in a specific region, with a median of 10 and standard deviation of 8.26. In some instances, the low numbers of species may be reflective of the fact that recreational teas were not the main object of some of our selected studies, or this could also be explained by differences in sample size and magnitude of the various studies. For example, one of the studies from Poland, PL1, with a high CI and the largest variety of species (37 taxa), was completed by scientists with special interest in plants used for making food-side tea. Nevertheless, our review has revealed that a specific cultural difference in the overall approach to selecting plant sources for recreational teas exists.

#### ***Russian Federation and other Slavic-speaking territories***

A rather low level of plant diversity used for making recreational tea was reported systematically among regions from the present Russian Federation. Moreover, many ethnographic publications describing the use of plants in the Slavic-speaking territories mention only one or two species used for making tea (e.g., see [55-58]). This might be related to the wide popularity of oriental tea (*Camellia sinensis* (L.) Kuntze) and its ceremonial use in these territories. Furthermore, in some of the Slavic- speaking territories, local plants are often used primarily as additives to oriental tea, and not as independent species used for making infusions (see the regions marked with * in Table 1). This pattern also extends beyond the territory of Russia. For example, Russian Old Believers that have lived within the borders of Estonia since the end of 17th century (region ES3), have persistently maintained this tradition (although they have adopted several local plants) and used them often as an addition to oriental black tea or in times of need. Prior to the introduction of oriental tea in Europe, one species, *Epilobium angustifolium* L., was considered to be the “original Russian tea” and was widely used throughout the country and abroad [59]. It has been recommended in many booklets and articles on tea-surrogates since the days of Linnaeus in Scandinavia [47,60,61].

#### ***Iberian Peninsula***

There are also great differences in the cultural importance of recreational teas among different areas. The higher values were found in Montesinho, in the northeast of Portugal (CI: 2.74, 21 species) [31,41]. Aromatic herbs were very important for the local gastronomy of the area. They were used for seasoning salads, soups, meat or sweet dishes, preparing herbal teas and liqueurs. Herbal teas were drunk hot in winter or cold in summer, as a refreshment. People liked their aroma and taste, and drunk them daily at any time as coffee substitutes*. Melissa officinalis* L., *Tilia platyphyllos* Scop., *Foeniculum vulgare* Mill. or *Calamintha nepeta* (L.) Savi were among the most salient species [41]. The hot meal of the day was usually rye bread and a soup made of boiled water with a few vegetable leaves, enriched with a tablespoon of rye, and seasoned with a great variety of aromatic herbs (e.g., *Mentha aquatica* L.*, M. suaveolens* Ehrh.*, M. x piperita* L.*, M. pulegium* L. or *Glechoma hederacea* L.). This variety of species offered people a variety in flavours despite the monotonous form of their core diet [34].

The lowest CI values were found in the Spanish Basque Country (CI: 0.02, 1 species). There, only one species was documented, *Chamaemelum nobile* (L.) All., reported by only two informants [37]. A possible explanation for this could lie within the cultural context of this region since the Basque people, as a pre-Indo-European ethnic group, have marked linguistic and cultural differences with the surrounding Latin regions. In fact, Basque traditional society has been historically impervious to innovations and new customs. The use of herbal teas in a food context is locally considered a modern tendency related to the introduction of oriental teas in Europe. Therefore, the spread of this new custom in a closed and traditionalist society can be more difficult. In study interviews, many people reported that herbal teas were only used in a medicinal context, and that those herbal teas taken like a coffee after meals were modern and not common in the region until recent years. Similarly, there was a rejection to spices and condiments in general [37] and people reported that they were commonly used only by immigrants, especially those from the south of Spain. In the rest of Spain, recreational teas are quite popular. This difference could be related to the Arabic influence, which was very weak in the Basque region.

#### ***Italy***

Ethnobotanical studies in Southern Italy report that teas in the classical sense (prepared as infusions) are not very popular. Instead, elderly people are more likely to prepare their hot beverages as decoctions, generally made by mixing dried herbs, fruits, and even cereals [62]. Decoctions, not teas, were in fact the traditional way that herbs were prepared and drunk in the Mediterranean. This continued practice may support the idea that in Southern Italy, the processes which took place among rural classes in Spain and the Balkans - the popularization of “teas” using local herbs, following the trends coming from the urban middle and high classes - did not develop in the same way.

One reason for this difference may be linked to the fact that in Italy, the Arabic or Turkish influences are scarce, and these were surely crucial in this process in Spain and in the Balkans. However, even in Sicily - the most Arab-influenced part of Italy – decoctions remained the most popular herbal drinks among rural classes [63]. The only place in Italy where the use of teas is remarkably popular is the Waldensian valleys in Piedmont (AP, unpublished data) and the surrounding Occitan valleys. Here, probably because of the continuous historical ties of the population in the last Centuries with their religious Protestant counterparts in France and especially in England, even poor mountain people became accustomed to taking a break in the afternoon for their “tea time”, generally using black tea or, more commonly, a rare local plant as a substitute: *Veronica allionii* Vill. [64].

### Substitutes for oriental tea

The importance of recreational tea compared to oriental tea has changed over both spatial and temporal planes. Before the oriental tea was imported to Europe, it was the only option and later served as a cheap local substitute for an expensive imported good in the 19th century and a healthy and nationalistic attitude before WWII. The author of a Polish 18th century economic plants dictionary, *Dykcyonarz Roślinny* “Plant Dictionary”, the priest Jan Krzysztof Kluk (1739–1796) created a long list of tea substitutes and his writing strongly opposed the use of oriental teas, explaining that Chinese tea “is packed into crates with the workers’ “bare feet”, and it is better to drink local, hand-picked herbal infusions [65].

Before the 1960s, tea was not regularly consumed in the Scandinavian countries. It was usually restricted to the upper classes and intellectuals. Only in some parts of western Sweden tea was also consumed by workers and peasants. Instead, Scandinavians were more apt to drink coffee [66]. Although tea was accepted as a beverage in the upper classes in Scandinavia already in the early 18th century, it was still an imported item. The economic policy in the early 18th century was to try to reduce the levels of imported tea. According to the government authorities, it was a luxurious imported product that could be substituted with native wild plants. For instance, in 1746 the Swedish authorities published a list of 45 plant taxa, mostly native species, which could be used as a substitute for tea and coffee [67]. Many floras also listed tea substitutes (e.g. [60]). During times of war, substitutes for imported products like oriental tea were widely recommended in many publications in Scandinavia. Moreover, many books have been published in Sweden since the mid-18th century suggesting substitutes for tea, such as the leaves of *Veronica chamaedrys* L. (1737), *Veronica officinalis* L. (1737), *Ligustrum vulgare* L. (1763) and *Rubus arcticus* L. (1886), or tea of *Rosa* spp., which were also used during World War II [13].

In the beginning of 20th century, when oriental tea finally became widely available in Estonia, many newspaper articles and books suggested a poor impact of oriental tea on one’s health and advised readers to use local species instead. This, along with the still high price for imported oriental tea resulted in the wide use of local species in official institutions (i.e. military and hospitals) and a relatively small level of consumption of oriental tea and coffee in the region [7].

Nowadays, oriental tea is available in many stores across Europe and its benefits for one’s health have been scientifically proven (e.g., see [68,69]). The status of recreational tea is dependent on access to the natural resources, cultural and social context, the habit of its use in the region, but most of all on the personal preferences of the consumer. For example, in Spain there are many people who prefer local teas to commercial *C. sinensis* teas. In fact, despite the loss of many wild plant uses, there are still a few recreational teas widely used and even served in restaurants (e.g., *Jasonia glutinosa* (L.) DC. or *Sideritis hyssopifolia* L.) [6]. While the variety of species is considerably large, the limited number of species used in several regions allows for some discussion regarding the preferred properties of the taxa used for making herbal recreational teas.

### Taste, smell and appearance

Some of the most important criteria concerning food preference include the taste, smell, and appearance [32,51,70-73]. Mild taste (in the opinion of researchers) has been shown to be the one criterion of selection for recreational teas in Estonia [7]. The sense of taste is very personal. The taste of an infusion depends greatly on the concentration of the plant and the mode of preparation. The majority of the most used plants have the taste and smell defined as being rather pleasant in given cultural settings, which in certain cultures is related to mild or fruity flavours, in others cases to aromatic teas and in some regions can even refer to bitter teas (MP, unpublished data). Whereas with regards to medicinal teas, plants are often considered to be very bitter or even unpalatable, a general requirement of a recreational tea must include a pleasant taste and smell to be attractive to all the potential drinkers. Also, in some regions, the colour of the infusion plays an important role in the preference of a particular recreational tea. For example, in Ukraine some interview participants preferred intensive colours and red was highly valued (IKD, unpublished data).

Unfortunately, to the best of our knowledge, there are no scientific studies that have been conducted concerning the lay perception of taste, smell and colour of the recreational teas made from the most popular species in either a specific cultural context or as an international comparison. While we could assess the descriptions of tea characteristics provided in the literature, this information is relatively scarce and difficult to analyze comparatively as descriptions of those characteristics have not been historically deemed important and are extremely rarely provided. Hence, more research is needed to address the question how the tastes, smells and colours of recreational teas made of most popular species are perceived and described by consumers and how they vary in different cultural settings. In Spain, the colour of most recreational teas varies from yellow- green to mild orange. In Madrid, the participants in recreational tea tasting trials have shown preference for intense golden colour and aromatic tastes (LA, unpublished data).

### Medicinal use and safety issues

As shown in the examples of herbal teas in Estonia, Spain and Portugal, the majority of plants used are perceived as medicinal plants in local folk medicine [7,34]. Whether the use of teas originated from the medicinal infusion or not, in modern practice it is not always easy to differentiate between a recreational tea and a tisane having medicinal value. Teas are indeed generally consumed on a daily basis within a food context, while medicinal infusions/tisanes are taken for a specific medical purpose. While medicinal herbal teas are purposely consumed for a limited number of days to treat a specific condition (i.e. cough, intestinal upset, etc.), there is no limit to the duration that recreational teas are consumed as they are used within a food context, and not for the treatment of medical conditions.

However, very often in rural Europe - especially in the south – home-made infusions and decoctions are prepared and drunk within the domestic domain on a regular basis, because they are considered to be “healthy” or because they are believed to prevent onset of certain illnesses. This “grey area” represents a very specific intersection of the food and medicinal domains, defined by Pieroni and Quave [74] as “folk functional foods”, being a serious obstacle to the popularization of recreational teas (in Italy), as drinking of such teas is perceived as prevention or treating, not recreational activity. Nevertheless, for example *Tilia* spp. being one of the most popular recreational tea genera since the 1930s in Estonia [7], is also the most used native taxon for complementary treating of common cold and flu in modern Estonia [75].

Moreover, the perceptions of a proper medicinal value of a tea, as well as its eventual “healthy” or merely recreational characters may change within the same community, or family, or even within the life history of the same person, depending on different situations/mood. Plants used for making recreational tea could also have a simultaneous use as a medicinal tea, while the degree of the overlap may differ greatly depending on the taxa [76]. To better understand this phenomenon, future field studies will need to pay close attention to the perceived medicinal and preventive properties of beverages as well as the frequency and variability of their use within the same study area.

Although a wide variety of species used in every region can serve as a guarantee for the variation of species used on an everyday basis, the safety issues related to long-lasting consumption of one particular local taxon cannot be underestimated. For example, some studies have evaluated the antioxidant and antiphrastic properties of essential oils and aqueous infusions of *Origanum vulgare* L. and *Thymus serpyllum* L. [77,78], and still, the safety of their everyday use is not proven. It is notable that in local herbals (e.g. [79,80]) both species are suggested to be used as medicinal plants or spices only and are not recommended for pregnant women, while their use as recreational tea, regardless their high popularity in Estonia [7], is not discussed at all.

## Conclusions

This review provides an assessment of the uses of local plants for the purposes of recreational teas throughout southern, central (although poorly represented) and eastern Europe over the past century. The results clearly indicate that most regionally important taxa are also important on the European or at least area level. Although the vast majority of the dominating species have already randomly been named among the plants used for making tea in different parts of Europe, such a comprehensive list of the most popular taxa has never been published before.

We can conclude that for European food culture, the most important families are Lamiaceae followed by Rosaceae in eastern and central Europe only and Asteraceae in all areas. On the genus level, the most important taxa are *Mentha*, *Origanum* and *Thymus*. On the species level, the overall favourite is *Origanum vulgare* L., followed by regionally important *Rubus idaeus* L. and *Thymus serpyllum* L. in eastern Europe, *Rosa canina* L. and *Hypericum perforatum* L. in central Europe, and *Chamaemelum nobile* (L.) All. in southern Europe.

Future research on the pharmacological, nutritional and phytochemical properties of the most popular plants used for making tea is important to ensure the safety and appropriateness of their use, especially as many of these are consumed on a daily basis. Moreover, in depth regional studies dedicated specifically to the use of local plants for making recreational teas will be important for developing a better understanding of their selection criteria, cultural importance and perceived properties in Europe and abroad.

## Expeditions

**Exp BG1** = Ethnographic and folklore expedition to Chepelare community of Smolyan region, Bulgaria. 2007. The expedition was organized by the Association of Anthropology, Ethnology, and Folklore “Ongal”, Sofia, Bulgaria.

**Exp BG2** = Ethnographic and folklore expeditions to L”ki community of Asenovgrad region, Bulgaria. 1992–1999. The expeditions were organized by the Association of Anthropology, Ethnology, and Folklore “Ongal”, Sofia, Bulgaria.

**Exp RO1** = Ethnobotanical expeditions to Pojana Mikuli village, Bukovina region, Romania 2005 – 2006.

**Exp RU1** = Ethnographic and folklore expeditions to St Petersburg region, Russia, 2000. Valeria Kolosova’s personal archive.

**Exp UA1** = Ethnographic and folklore expeditions to the village Stari Broskivtsi, Storozhinets region of Сhernovtsy oblastj, Ukraine, 1999–2000. The expeditions were supported by European University at Saint-Petersburg and the Individual Research Support Scheme Grant “Flowers and Herbs in Ukrainian Traditional Culture” (RSS No. 1140/2000). Valeria Kolosova’s personal archive.

**Exp UA2** = Ethnobotanical expedition to Strointsy village, Tivriv region, Vinnitsa oblast, Ukraine’ march-August 2012. Research supported by Polish Ministry of Education, NCN grant number: 2011/01/N/HS3/03332.

## Competing interests

The authors declare that they have no competing interests.

## Authors’ contribution

RS – initiated the article, analyzed regional data, wrote first draft and finalized the article. CQ – provided data on IT1-2 and SE1 and thoroughly edited the article. AP – provided data on IT1 and SE1 and substantially contributed to data analysis and the discussion. RK – provided data on EE1-4 and contributed substantially to the discussion. ŁŁ provided data on PL2 and edited different versions of the article. IS contributed by providing and discussing general history and Scandinavian data. VK provided data on RU1-4, UA1, BG1-2. MPS, LAM, GM and JT provided data on SP1-6 and PT1 and contributed substantially to the discussion and data analysis. EP provided data on PL1 and contributed to data analysis. IKD provided data on UA2 and RO1. RP provided data on LT1 and BY1. AH and BM provided data on KS1-2. All authors have contributed to and approved the manuscript.

## Supplementary Material

Additional file 1 Aggregate table containing all details (including species not listed in the tables and URs for every specific regions). Click here for file
